# Bioparticles coated with an ionic liquid for the pre-concentration of rare earth elements from microwave-digested tea samples and the subsequent quantification by ETV-ICP-OES†
†Electronic supplementary information (ESI) available. See DOI: 10.1039/c6ay02189a



**DOI:** 10.1039/c6ay02189a

**Published:** 2016-10-10

**Authors:** Sara Hosseinzadegan, Winfried Nischkauer, Katharina Bica, Andreas Limbeck

**Affiliations:** a Institute of Chemical Technologies and Analytics , TU Wien , Getreidemarkt 9/164-IAC , 1060 Vienna , Austria . Email: Andreas.Limbeck@tuwien.ac.at; b Institute of Applied Synthetic Chemistry , TU Wien , Getreidemarkt 9 , 1060 Vienna , Austria

## Abstract

An analytical procedure for straight-forward quantification of rare earth elements (REEs) in tea was developed. The method consists of three steps: first, dry tea powder is converted into an aqueous sample solution using a microwave-assisted digestion procedure. Then, the REEs are retained on newly designed sorbent particles and are thus effectively extracted from the sample digest. Then, the REE-loaded sorbent material is introduced into the furnace of an electro-thermal vaporization (ETV) unit. There, the core of the particles is pyrolyzed and removed in a first temperature step. Then, the REE analytes are swiftly evaporated at high temperatures, and subsequently analyzed by ICP-OES. The advantages of the method proposed here are as follows: extraction of REEs is accomplished *via* “dispersed particle extraction”; this avoids typical shortcomings of conventional solid-phase extraction. The analysis *via* ETV allows separation of the sorbent particles from the analytes in time. Thus, detrimental effects of plasma-loading are circumvented. The method was shown to provide satisfactory detection limits of typically 50 ng g^–1^ in the dry tea samples (3 s criterion). The method was validated by means of spike recovery experiments, and applied to the analysis of ten different tea samples from China, Japan and India, as sold in European markets.

## Abstract 2

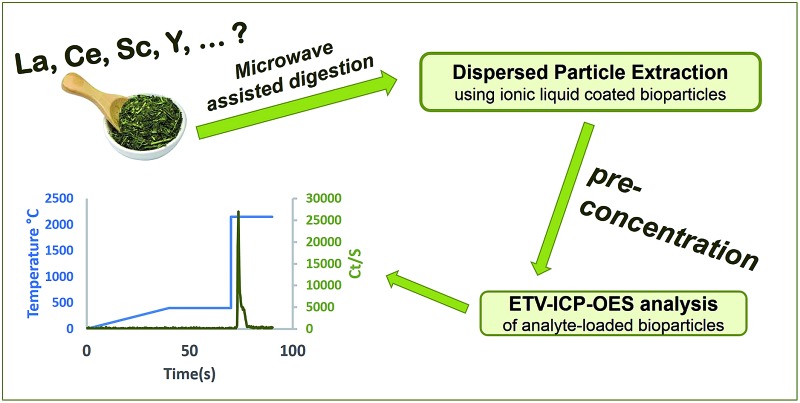
An analytical procedure for straight-forward quantification of rare earth elements (REEs) in tea was developed.

## 1. Introduction

Rare earth elements (REEs) are a group of elements which include fourteen lanthanides plus scandium and yttrium. Although they are named rare, their abundance in the environment is comparable to that of other heavy elements such as Cu, Ni, Co and Zn. Recently, there has been increased tendency to apply REEs in several fields which include agriculture,^1^ catalysts^2^ and cell phones.^3^ The increased industrial use of REEs results in pollution of the environment, followed by accumulation of these elements in the biosphere and this accumulation causes an increased risk for human health. Based on reports, accumulation of REEs^4^ could cause serious problems in different organs of the human body^5^ especially in bones^6^ and the nervous system.^7^ During plant growth, toxic elements present in soil or water can be taken up by plants together with essential elements. Tea made from *Camellia sinensis* leaves, both black and green, is a common and popular non-alcoholic drink all around the world. Accordingly, it seems that the level of REEs should be monitored in tea. Until now, data about REE levels in tea are very sparse, and there are only a few studies published^8–11^ that indicate that the level of REEs especially La, Ce, Y, Pr and Nd in tea is considerable.

For analysis of trace elements in environmental samples, the most appropriate techniques include inductively coupled plasma mass spectrometry (ICP-MS), inductively coupled plasma optical emission spectrometry (ICP-OES) and electro-thermal atomic absorption spectrometry (ET-AAS).^12^ However, accurate analysis of REEs in plant samples is challenging – REE levels in the range of a few ng g^–1^ up to some 100 ng g^–1^ combined with numerous interferents in the most sensitive analytical techniques are the major difficulties. To overcome limitations in sensitivity and/or selectivity (*e.g.* due to spectral interferences), the use of sample pre-treatment procedures for pre-concentration and quantitative separation of REEs from interfering sample constituents is recommended. Although several enrichment processes have been reported in the literature, solid phase extraction (SPE) has received the most attention due to easy automation and being inexpensive. In SPE, the analyte-containing matrix is passed through a column of an adsorbent on which the analytes of interest are retained. After a washing step to remove the remaining matrix, the analytes are eluted with an appropriate solvent. Thus, besides analyte enrichment, an effective removal of the potentially interfering matrix can also be achieved. Nevertheless, this way of enrichment has some disadvantages including time demand, column aging and large sample and solvent consumption.^13^

Regarding the aforementioned disadvantages of SPE, our group has recently introduced an improved approach called dispersed particle extraction (DPE). In this approach, adsorbent particles with surface modified functionalities are suspended in a liquid sample. Targeted analytes are trapped by the particles while the matrix remains in solution. After centrifugation, the supernatant solution is discarded, the sedimented particles are diluted with acid and finally the suspension is measured by a slurry ICP-OES procedure.^14^ To overcome the main disadvantage of slurry analysis (potential nebulizer clogging), the sorbent particles can also be dissolved with the use of mineral acids, followed by conventional liquid ICP-MS analysis of the derived solution.^15,16^ However, the dissolved sorbent material still increases the plasma load during ICP-MS measurement, which could affect atomization, excitation and ionization of the target analytes in the plasma.

In this work, an innovative approach is presented, which is based on enrichment of REEs using DPE, followed by sensitive measurement of the analyte-containing sorbent particles by means of electrothermal vaporization (ETV) in combination with ICP-OES detection. ETV allows to first pyrolyze the adsorbent material, and only then to introduce the REEs into the ICP. In this way, an effective separation of the particle matrix and analytes in time is enabled, thus plasma load from vaporization of the particles could be avoided during analyte measurement. Since the thermal behavior of REEs and commonly applied silica particles^14–16^ is quite similar, DPE has been performed with organic sorbent particles in this work for the first time. The applied adsorbent is a natural bio-particle with high porosity, *Lycopodium clavatum* spore, which was covered with a layer of ionic liquid. The ionic liquid acts as the cation exchanger. These natural plant spores with an average size of 30 microns are an excellent choice as a core material because they are stable in strong acids or bases, inexpensive, easily approachable and ecological.^17^ Even though the particles are very porous, they do not swell in different solvents.^18^ The applicability of the method is demonstrated by the analysis of REE levels in different green tea samples.

## 2. Experimental

### 2.1 Reagents and standards

High purity water (18 MΩ cm) was prepared with an Easypure purification system (Thermo, USA) and was used in the whole process. Nitric acid 65% (Merck), hydrochloric acid 37% (Merck), hydrofluoric acid 40% (Merck), ethanol 99% (Chem-Lab) and acetone 99.8% (Acros Organics) purchased were of p.a. grade. Acetic acid 96% (Merck) and ammonium acetate (Merck) were used for buffering the samples prior to extraction. Single element ICP standards including La, Eu and Nd (Aristar, 1000 mg L^–1^), In (Merck, 1000 mg L^–1^) and a multi-element REE calibration standard (Agilent Technologies, multi-element calibration standard 1, 10 mg L^–1^, 5% (v/v) HNO_3_) were diluted to the concentration needed.

A set of ten different green tea samples was bought at a local market in Vienna (details on each tea are summarized in Table 1). Prior to chemical digestion, the samples were dried for 24 h at 60 °C.

**Table 1:** Description of tea samples

Sample ID	Origin	Description
Tea 1	China	Gunpowder green tea
Tea 2	India	Organic green tea
Tea 3	Fujian, China	Organic Tiegunyin tea
Tea 4	China	Oolong tea
Tea 5	China	Chun-Mee
Tea 6	China	Sencha
Tea 7	China	“Teekanne” green tea
Tea 8	Japan	Genmaicha
Tea 9	China	Fresh green tea
Tea 10	China	“Grüner Kimono”
Tea 11	China	Fresh green tea

### 2.2 Instrumentation

ETV-ICP-OES measurements were performed using radial ICP-OES (Thermo iCAP 6500, Thermo Scientific, USA), coupled to an ETV 4000 unit (Spectral Systems, Germany). The plasma torch contained a corrosion-resistant ceramic injector tube of 1.5 mm inner diameter and was connected to the ETV *via* 40 cm of PTFE tubing of 4 mm inner diameter.

For conventional ICP-OES analysis of liquid samples, a sample introduction kit consisting of a Babbington (V-groove) nebulizer, a glass cyclonic spray chamber with a riser tube and a torch injector tube with 2 mm inner diameter was used.

Digestion of tea leaves was performed by microwave-assisted closed vessel treatment (Multiwave 3000, MF 100 vessels, Anton Paar, Austria).

Separation of particles during the enrichment process was performed by using a Megafuge 16 Centrifuge (Thermo Scientific, Germany). The centrifuge was equipped with a FIBERLite F15-6 × 100 angular rotor. The sample pretreatment procedure was carried out in 15 mL conical polypropylene centrifugation tubes with a V-bottom (maximum tolerable acceleration: 17 000 × *g*, VWR, Germany).

### 2.3 Preparation and characterization of sorbent particles

The ionic liquid used for the extraction of REEs consisted of the trihexyl(tetradecyl)phosphonium cation and the di(2-ethylhexyl) phosphate anion (see Fig. S1, ESI†). Throughout this article, the nomenclature [P_66614_]^+^[BEHPA]^–^ will be used for the ionic liquid. Synthesis of [P_66614_]^+^[BEHPA]^–^ was carried out at the Institute of Applied Synthesic Chemistry of TU Wien, according to the following scheme: trihexyltetradecylphosphonium chloride (5.19 g, 10 mmol), bis(2-ethylhexyl) hydrogenphosphate (3.54 g, 11 mmol) and potassium hydroxide (0.62 g, 11 mmol) were suspended in 100 mL of water : MeOH 1 : 1 and stirred for 2 h at room temperature. The solution was concentrated to approx. 60 mL and diluted with water. This mixture was repeatedly extracted with dichloromethane, and the combined organic layers were washed with water until no more chloride ions could be detected (checked by addition of an aqueous AgNO_3_ solution). The organic phase was dried over Na_2_SO_4_ and concentrated under reduced pressure. Remaining solvent traces were removed under high vacuum (0.01 mbar, 48 h) with stirring at 50 °C to yield trihexyltetradecylphosphonium bis(2-ethylhexyl)phosphonate as a clear colorless liquid in 94% yield. The derived product was characterized by NMR (see ESI†).

To make a cation exchange adsorbent, 990 mg of *Lycopodium clavatum* spores were added to the solution of 10 mg of ionic liquid [P_66614_]^+^[BEHPA]^–^ in 50 mL ethanol in a 100 mL round bottom flask. The mixture was shaken smoothly on a slow-moving platform shaker for 2 hours at room temperature. Ethanol was subsequently evaporated using rotary pump vacuum and the solid product was dried overnight under high vacuum.

**Fig. 1:**
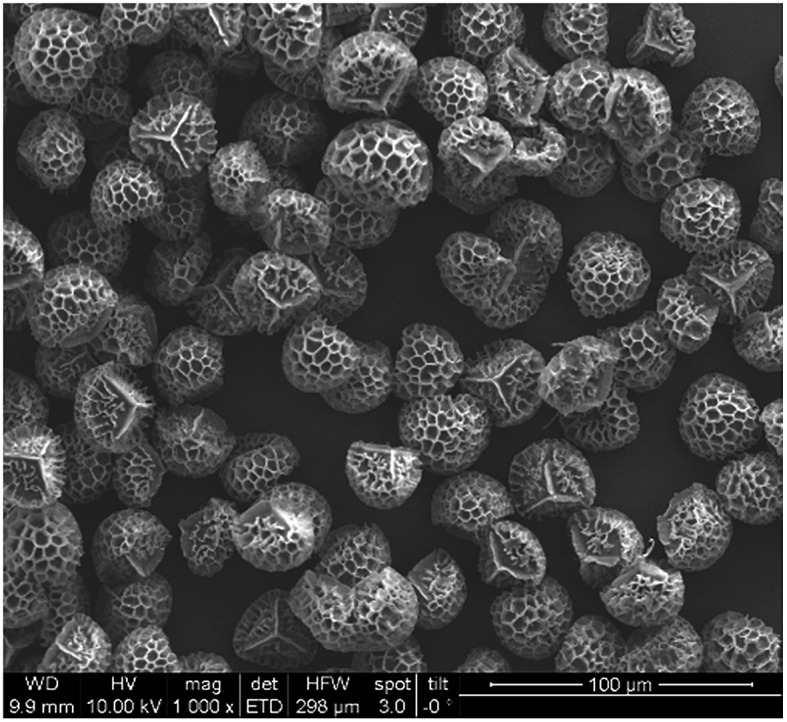
SEM images of [P_66614_]^+^[BEHPA]^–^@*Lycopodium clavatum*, 10 kV acceleration voltage and 100 μm scale-bar.

Particles were characterized using scanning electron microscopy (Quanta 200 MK2, FEI, USA) in the initial form and after coating with the ionic liquid. The SEM images of raw *Lycopodium clavatum* and the treated spores indicate no change in the morphology after treatment. As is shown in Fig. 1, the coated particles have a size of roughly 30 microns.

### 2.4 Microwave assisted sample digestion

For complete decomposition of tea, 0.6 g of the dried leaves were transferred to clean microwave vessels and digested in a mixture of 3 mL HNO_3_ (65 wt%), 2 mL HCl (37 wt%), 1 mL H_2_O_2_ (30 wt%) and 0.1 mL HF (40 wt%) under elevated pressure and temperature: 40 min heating ramp, 30 min holding time at 800 W power, a maximum temperature of 190 °C and a maximum pressure of 20 bar. The obtained clear solutions were quantitatively transferred into PFA beakers (Nalgene, USA) and evaporated to dryness on a heating block at 140 °C. The dried residues were treated for another 30 minutes with a mixture of 2 mL HNO_3_ (65 wt%) and 2 mL H_2_O_2_ (30 wt%) at 90 °C with closed caps. Then, the solutions were again evaporated to dryness to remove any remaining HF.^19,20^ The residues were dissolved with 50 μL of 0.07 M HNO_3_ in an ultrasonic bath (5 min) and then filled up to 35 mL with high purity water.

### 2.5 Dispersed particle extraction procedure

**Fig. 2:**
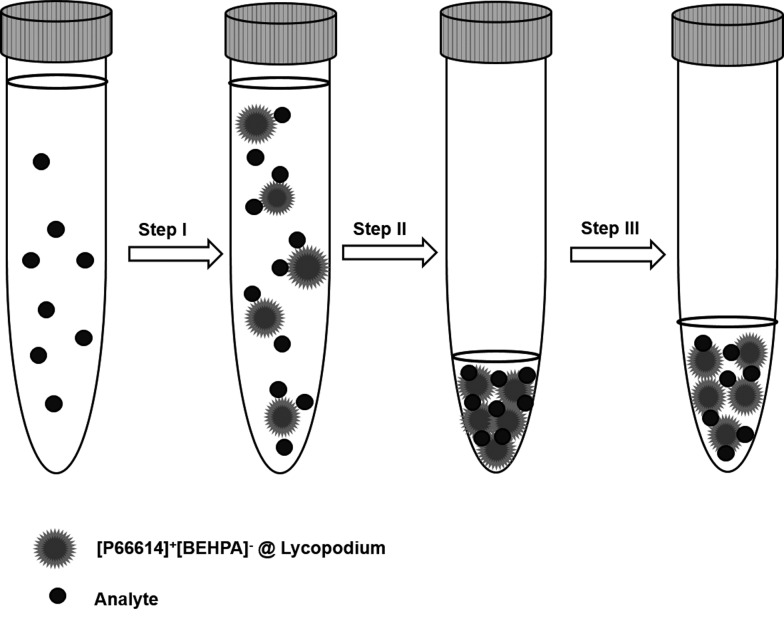
Illustration of the dispersed particle extraction (DPE) enrichment process.

A graphical illustration of the developed sample pre-treatment procedure is presented in Fig. 2. For analysis 10 mL of sample solution was pipetted into 15 mL conical tubes, and ammonium buffer was added for pH adjustment. Dry sorbent particles were added to the sample solution (step I). After a reaction time of 5 minutes, necessary to retain the REE ions on the sorbent particles, the sample solution was centrifuged for the separation of solution and analyte-containing particles. After removal of the supernatant solution (step II), the precipitated sorbent particles were re-suspended in dilute nitric acid (1% v/v) to obtain a final volume of 500 μL in the case of ETV experiments (step III). For conventional slurry ICP-OES measurement, the volume was adjusted to a final volume of 2 mL. During step III, indium was added as the internal standard with a final concentration of 6 mg L^–1^.

### 2.6 Sample analysis

During method development, the slurry obtained after DPE extraction was analyzed *via* slurry nebulization ICP-OES. Measurement was done with the use of a Babington nebulizer with a sample flow rate of 0.6 mL min^–1^. Background-corrected signals were recorded in 5-fold replicates using an analysis time of 5 s per replicate.

For the final ETV-ICP-OES method, 30 μL of the suspension obtained *via* DPE was transferred into a cleaned graphite boat. Before measurement, the solvent was evaporated by gentle heating with an infrared lamp. Then, the boat was placed in the ETV oven and heated following a defined program for 90 s. The boat was first heated to 400 °C by a slow ramp in 40 s and was then held at that temperature for 30 s. Then, it was swiftly heated up to 2150 °C and was held for 20 s at that temperature, followed by a cooling phase. The instrumental parameters of the applied analytical procedures are summarized in Table 2. Since the resolution of the time-axis is inversely proportional to the number of observed emission lines, only one emission line per element was selected. With those settings, “Timescan” mode was used to record the derived transient signals by collecting data points every 0.4 s.

**Table 2:** Instrumental parameters used for conventional and ETV measurement and analytical wavelengths

		ETV sample introduction	Liquid sample introduction
Plasma power	W	1200	1200
Radial observation height	mm	11	11
Plasma gas (Ar)	L min^–1^	12	12
Nebulizer gas (Ar)	L min^–1^	—	0.9
Auxiliary gas (Ar)	L min^–1^	0.8	0.8
Freon R12 modifier	mL min^–1^	10	—
Bypass gas (Ar)	L min^–1^	0.38	—
Carrier gas (Ar)	L min^–1^	0.14	—

Element	*λ* (nm)	Element	*λ* (nm)
Ce	415.197	Nd	430.358
Dy	364.540	Pr	390.844
Er	369.265	Sc	361.384
Eu	381.967	Sm	360.949
Gd	364.619	Tb	350.917
Ho	339.898	Tm	342.508
La	333.749	Y	371.030
Lu	261.542	Yb	369.419
In*	325.609	* Indium was used as the internal standard	

## 3. Results and discussion

### 3.1 Optimization of the extraction process

To optimize the DPE procedure for REE retention on [P_66614_]^+^[BEHPA]^–^@*Lycopodium clavatum*, several experiments were performed to find optimal conditions in terms of the sample pH, amount of sorbent material, and reaction time. These preliminary experiments were done with solutions containing 100 μg L^–1^ of only three elements (Eu, La and Nd). Measurement of derived sample slurries was performed using conventional liquid ICP-OES analysis.

As in most cation-exchange procedures, sample pH is one of the most important factors. In preliminary experiments, the pH range 2–6 was tested by adjusting the pH with different concentrations of ammonium acetate buffer, and the maximum retention for REEs was obtained for the sample solution of pH = 5.

In contrast to column-based SPE approaches, the sorbent is directly added to the analyte solution in the DPE procedure applied here. Therefore, optimization should be done to ensure that enough sorbent material is available for retaining the target elements – a prerequisite for reproducible analysis. On the other hand, an excess amount of particles requires a slower heating ramp during ETV analysis, in order to avoid extinguishing the ICP due to the sudden formation of huge amounts of CO_2_. Therefore, the addition of 1, 2.5, 5 and 10 mg of the particles to 10 mL sample solution was examined and the results showed that 5 mg adsorbent material was sufficient to obtain around 80% ± 3% recovery for Eu, La and Nd.

Another parameter affecting the extraction process is the reaction time. To find the optimum extraction time, 10 mL samples of the 100 μg L^–1^ analyte with 5 mg particles and pH 5 were shaken smoothly by hand for 2, 5, and 10 min. Accordingly, 80% recovery for Eu, La and Nd can be obtained after 5 min reaction time and a longer time does not improve the analyte retention. It should be mentioned that ultrasonic agitation has a negative effect on the recovery because it probably leads to the release of the ionic liquid from the surface of the *Lycopodium* particles.

Summing up, the ideal conditions for REE extraction (investigated for Eu, La and Nd) are: pH = 5, amount of adsorbent particles = 5 mg, and extraction time = 5 min.

### 3.2 ETV-ICP-OES measurement

First, the ETV program was optimized for best performance in terms of matrix pyrolysis and analyte-release. To avoid extinguishing the plasma by the rapid formation of CO_2_ (decomposition of the sorbent particles), a heating ramp from room temperature to 400 °C during 40 s, followed by a plateau of 30 s at this temperature was found to ensure stable plasma conditions. Lower pyrolysis temperatures resulted sometimes in incomplete sample pyrolysis, which resulted in extinguishing the plasma during the following vaporization step, whereas higher pyrolysis temperatures can result in analyte losses. The vaporization temperature was optimized in terms of quantitative analyte release, as well as in terms of graphite-tube life-time (too high temperatures result in a reduced life-time of the tube). In order to vaporize refractory elements such as Y, a gaseous modifier (Freon R12) was added to the carrier gas. Under optimized conditions, a second measurement of the same boat resulted in signals equal to the blank of an empty boat.

**Fig. 3:**
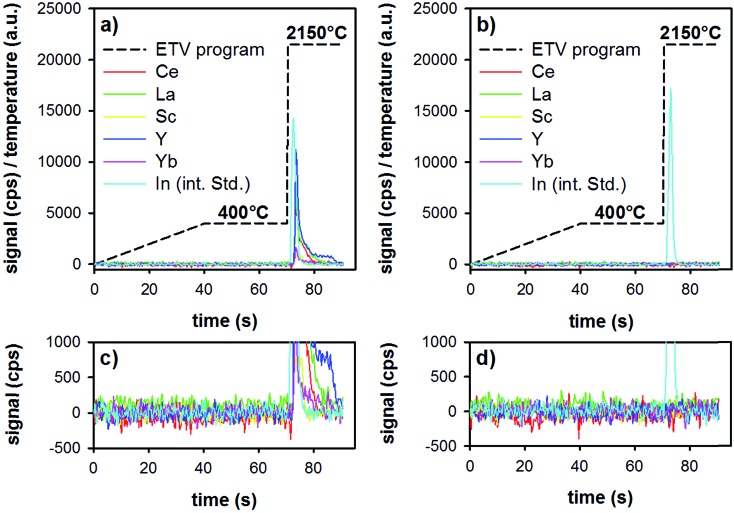
Heating program (dashed black line) used for ETV-ICP-OES analysis and the recorded signals for Ce, La, Sc, Y, Yb, and In (as the internal standard) in a tea sample pre-treated by the method presented here (a and c), and in a method blank (b and d). ((c and d) are enlarged versions of (a and b), to see the background situation more clearly).

ETV analysis of samples was carried out as follows: in the first step, the loaded boat was heated up to 400 °C in 40 s, then was held for 30 s at that temperature. This step allows the slow pyrolysis of the organic material (*Lycopodium* particles and ionic liquid) present in the investigated sample slurry, thereby a separation from the less volatile REEs could be accomplished. Then, by increasing the temperature to 2150 °C with the maximum heating rate, only the remaining inorganic sample constituents including the target elements are introduced into the plasma. The whole heating program requires 90 s and the analyte signals are continuously recorded with the software of the ICP-OES spectrometer. The amount of sample volume added into the graphite boats was first optimized (10–40 μL), and was found to be optimal at 30 μL. Higher sample volumes resulted in detector saturation for some elements (*i.e.*, the highest spiked standard-addition samples). The temperature program and typical time-resolved analyte signals are illustrated in Fig. 3. The analyzed samples were a digested tea sample (Fig. 3a), as well as a method blank (all reagents, including microwave digestion, see Fig. 3b). Both samples were pre-treated with the above-mentioned DPE procedure and analyzed by means of ETV as described.

It is clear from Fig. 3 that during the first 70 s of the ETV program, there is no signal of the REEs or of the internal standard. The two insets (Fig. 3c and d) show an enlarged version of the background. This shows that the method blank is negligible, and that matrix pyrolysis does not lead to earlier release of analytes. Upon elevating the temperature to 2150 °C, analytes and the internal standard are evaporated smoothly, resulting in sharp transient signals. Repeated analysis of one boat showed that the analytes are released quantitatively already during the first firing of the ETV program.

### 3.3 Figures of merit

By applying the optimized parameters obtained for REE retention on [P_66614_]^+^[BEHPA]^–^@*Lycopodium clavatum*, the enrichment factor, reproducibility and the limit of detection were calculated for all investigated elements. For this purpose, an aqueous calibration row was prepared and these solutions were then treated with the discussed DPE approach. The reproducibility of measurement was determined by replicate pre-concentration and measurement (*n* = 4) of a sample solution containing 20 ng mL^–1^ of the investigated REEs, leading to relative standard deviations ranging from 4% (Nd) to 18% (Dy). Based on the conditions used for sample pre-concentration – 10 mL applied sample volume and 500 μL final volume – it is possible to achieve a theoretical enrichment factor of 20. By comparing the slopes of the calibration curves before and after pre-concentration, the actual enrichment factor was determined to be 17.8.

The LOD was calculated from the standard deviation of 12 blank measurements, according to the 3 s criterion. To obtain the LOD in the non-digested tea sample, a sample intake of 0.5 g dry tea, digested and diluted to a final volume of 35 mL was considered. As can be seen in Table 3, typical detection limits are around 50 ng g^–1^ in the dry tea samples.

**Table 3:** Method LOD in the case of ETV-ICP-OES (all data refer to concentration in the dry tea, ng g^–1^)

Element	Ce	Dy	Er	Eu	Gd	Ho	La	Lu
LOD	30	8	20	9	60	50	40	2

Element	Nd	Pr	Sc	Sm	Tb	Tm	Y	Yb
LOD	90	40	5	80	40	30	4	5

### 3.4 Influence of the sample matrix on DPE

**Fig. 4:**
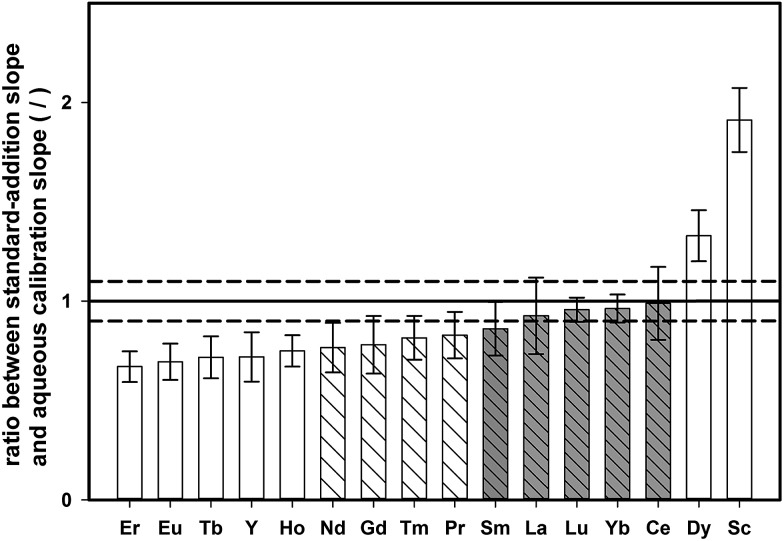
Ratio of the slopes obtained from standard-addition calibration and aqueous calibration (both in the range 0–10 μg L^–1^, the error corresponds to the expanded combined uncertainty of the ratio, *k* = 2, and the dashed lines correspond to ±10% deviation from a nominal ratio of 1).

In contrast to the synthetic aqueous standard solutions used for method development, real samples contain also the sample matrix, which might have an impact on the enrichment procedure. To study the effect of the digested tea matrix on the recovery of REEs, the slope obtained by standard addition onto digested tea (tea sample 4, see Table 1) was compared with the slope of aqueous standards. Both sets of samples were pre-concentrated with the described DPE approach and analyzed by means of ETV-ICP-OES. For each calibration curve, the standard uncertainty of the slope was also calculated. The ratio of standard addition slope to aqueous calibration slope for all the investigated elements is shown in Fig. 4, together with the corresponding expanded combined uncertainty (*k* = 2).

If there is no matrix effect, the ratio of both slopes is expected to be 1, which applies to Sm, La, Lu, Yb, and Ce. However, in the case of Er, Eu, Tb, Y, Ho, Nd, Gd, Tm, and Pr, the ratio is below 1, indicating that the slope of the standard addition curve was flatter than the slope of an aqueous calibration. This outcome indicates that the recovery of these elements is decreased in the presence of the tea matrix. On the other hand, Dy and Sc showed the opposite trend revealing higher signals for aqueous standards than for matrix-containing tea digests. However, when accepting a deviation within 10% of the nominal ratio of 1, most elements could be quantified with external aqueous calibration (Nd, Gd, Tm, Pr, Sm, La, Lu, Yb, and Ce).

For some elements, external calibration with aqueous standards might lead to under/overestimation of true contents. Matrix-adjusted calibration (*i.e.*, a blank-corrected standard addition curve) was investigated as an alternative quantification approach to provide correct results for all elements investigated. Although this approach showed good results when performing spike-recovery experiments on one single tea sample (tea sample 4), with recoveries ranging from 90% to 124%, the analysis of different types of green tea showed that analyte-recovery also slightly changes due to variations in the sample matrix. Therefore, the concept of two-point standard-addition was relied upon for correct quantification in all of the following experiments, since it is less work-intensive as matrix-matched calibration for every tea sample.

### 3.5 Analysis of tea samples

For method validation, one tea-sample (tea-sample 11) was digested, and analyzed also using quadrupole ICP-MS in the kinetic energy discrimination mode (KED-mode). Details regarding the ICP-MS method can be found in the ESI.† For this tea-sample, most elements were below the respective detection limits, but for three elements, it was possible to obtain quantitative data by both methods. For those values, good agreement between ICP-MS results (La: 720 ± 40 ng g^–1^, Ce: 410 ± 20 ng g^–1^, Y: 240 ± 20 ng g^–1^) and the proposed ETV-ICP-OES procedure (La: 700 ± 80 ng g^–1^, Ce: 440 ± 20 ng g^–1^, Y: 180 ± 20 ng g^–1^) was obtained.

Dispersed particle extraction was applied for enrichment of rare earth elements in ten different green tea samples (see Table 1), followed by measuring the pre-concentrates by means of ETV-ICP-OES. Quantification was carried out by means of standard-addition. To this end, one aliquot of the digested tea sample was pre-treated directly, whereas another aliquot was spiked with REEs and only then was subjected to the procedure. Table 4 summarizes the results of the investigated samples. For Ce, La, and Y all samples provided signals distinctly different from those of procedural blanks. The Gd and Tb contents were below the respective detection limits in all tea samples. For some elements (Dy and Eu), there was no significant variation observed in between the samples, whereas other elements (Ce and La) showed concentrations differing by up to a factor of five. The obtained concentrations are generally in good agreement with typical REE-concentrations in tea samples. According to the results of green tea reference material GBW07605 analysis by ICP-OES^21^, Ce, La, and Y have concentrations of 0.92, 0.57 and 0.37 μg g^–1^ in tea leaves. In another work regarding the determination of REEs in four different tea samples by using ICP-MS^8^ the range of concentration for Ce, La, and Y are respectively 0.62–0.99, 0.82–1.84 and 0.13–0.20 μg g^–1^. These values are in similar order to the concentrations obtained in our work for Ce, La and Y including 0.340–1.800, 0.390–2.000, 0.090–0.540 μg g^–1^ respectively.

**Table 4:** Measured REE-concentrations in 10 different tea samples (all values in ng g^–1^ in the dry tea, *n* = 2, error corresponds to the standard deviation)

	Ce	Dy	Er	Eu
Tea 1	340 ± 5	<8	<20	<9
Tea 2	850 ± 360	270 ± 10	1200 ± 60	40 ± 10
Tea 3	1500 ± 330	280 ± 30	800 ± 120	20 ± 5
Tea 4	1700 ± 180	290 ± 10	<20	<9
Tea 5	600 ± 130	<8	<20	<9
Tea 6	1800 ± 90	290 ± 10	880 ± 30	30 ± 5
Tea 7	480 ± 160	240 ± 15	840 ± 30	20 ± 10
Tea 8	180 ± 70	<8	<20	20 ± 5
Tea 9	1000 ± 280	230 ± 5	1300 ± 10	20 ± 5
Tea 10	850 ± 130	240 ± 5	<20	20 ± 5

	Gd	Ho	La	Lu
Tea 1	<60	<50	390 ± 10	10 ± 5
Tea 2		1300 ± 170	870 ± 440	20 ± 5
Tea 3		<50	1400 ± 400	20 ± 5
Tea 4		<50	2000 ± 100	10 ± 5
Tea 5		<50	780 ± 120	10 ± 5
Tea 6		830 ± 20	2000 ± 160	20 ± 5
Tea 7		870 ± 120	540 ± 80	20 ± 10
Tea 8		730 ± 100	370 ± 110	<2
Tea 9		1100 ± 70	750 ± 210	30 ± 20
Tea 10		<50	790 ± 110	20 ± 5

	Nd	Pr	Sc	Sm
Tea 1	<90	<40	<5	<80
Tea 2	7300 ± 480	<40	70 ± 5	1000 ± 120
Tea 3	5200 ± 400	260 ± 80	70 ± 5	<80
Tea 4	<90	<40	<5	<80
Tea 5	3700 ± 400	<40	30 ± 5	<80
Tea 6	5400 ± 250	180 ± 30	40 ± 5	<80
Tea 7	5000 ± 430	120 ± 10	50 ± 5	<80
Tea 8	4600 ± 330	<40	<5	<80
Tea 9	7800 ± 860	<40	50 ± 5	1000 ± 190
Tea 10	3700 ± 410	160 ± 5	40 ± 10	<80

	Tb	Tm	Y	Yb
Tea 1	<40	<30	140 ± 10	20 ± 5
Tea 2		810 ± 150	460 ± 130	60 ± 10
Tea 3		640 ± 100	240 ± 40	40 ± 10
Tea 4		<30	180 ± 5	30 ± 5
Tea 5		<30	160 ± 20	20 ± 5
Tea 6		670 ± 50	540 ± 10	50 ± 10
Tea 7		570 ± 20	400 ± 110	50 ± 10
Tea 8		440 ± 10	90 ± 10	<5
Tea 9		880 ± 20	460 ± 10	50 ± 5
Tea 10		490 ± 80	450 ± 80	60 ± 5

## 4. Summary and outlook

In this work, the concept of dispersed particle extraction has been further explored by ETV-ICP-OES for direct measurement of the analyte-containing sorbent particles. Compared to conventional slurry analysis, distinct improvements in sensitivity could be achieved; furthermore sample introduction by ETV allows the separation of the analyte and matrix in time. In order to achieve this goal, the use of an organic sorbent material instead of commonly applied silica based materials was necessary. For this purpose, *Lycopodium clavatum* spores coated with an ionic liquid acting as the cation exchanger were used, which could be completely volatilized during the matrix-pyrolysis step in the ETV program.

The method was applied to the analysis of REEs in digested tea samples, with typical detection limits around 50 ng g^–1^ in the dry tea. The measurement procedure was shown to produce good results in terms of analyte recovery when performing spike-recovery experiments in combination with matrix-adjusted calibration. However, since slight differences in the matrix between different tea-samples persisted, two-point standard addition was relied upon for quantification. The results obtained for ten tea samples are in the same concentration range as reported by other authors.^8,21^

The method presented here is a first proof of principle to show the applicability of natural bio-particles coated with ionic liquid for extracting and pre-concentrating REE elements. The method will be further optimized in terms of the pre-concentration factor as well as the digestion procedure, in order to mineralize the organic sample-matrix of the tea entirely. Then, it should be possible to rely upon aqueous external calibration, which would enhance the sample throughput.

Furthermore, the procedure could be easily adapted to other tasks by simply changing the ionic liquid used for analyte extraction. With the use of task-specific ionic liquids, improvements not only in sensitivity but also selectivity are expected, compared to conventional SPE methods.
