# Supplementary material for: Bioparticles coated with an ionic liquid for the pre-concentration of rare earth elements from microwave-digested tea samples and the subsequent quantification by ETV-ICP-OES
Source: Analyst. 2016 Oct 10;8(43):7808–15. doi: 10.1039/c6ay02189a (PMC5123633; doi:10.1039/c6ay02189a)
Supplement: Supplementary file 1 [file AY-008-C6AY02189A-s001.pdf]

Electronic Supplementary Material (ESI) for Analytical Methods.
This journal is © The Royal Society of Chemistry 2016

Bioparticles coated with ionic liquid for the pre-concentration of rare earth elements from microwave-digested tea samples and the subsequent quantification by ETV-ICP-OES

Sara Hosseinzadegan[a‡], Winfried Nischkauer[a‡], Katharina Bica[b], and Andreas Limbeck[a] *

[a] Institute of Chemical Technologies and Analytics, TU Wien, Getreidemarkt 9/164-IAC, 1060 Vienna, Austria.

[b] Institute of Applied Synthetic Chemistry, TU Wien, Getreidemarkt 9, 1060 Vienna, Austria.

* Andreas.Limbeck@tuwien.ac.at

Electronic Supplementary Material

Structure and characterization of $[P66614]^+[BEHPA]^-$

^1H and ^{13}C NMR spectra were recorded from $CDCl_3$ solutions on a Bruker Advance UltraShield 400 (400 MHz) spectrometer and chemical shifts (δ) are reported in ppm using tetramethylsilane as internal standard. Coupling constants (J) are in Hertz (Hz). The following abbreviations were used to explain the multiplicities: s = singlet, d = doublet, t = triplet, q = quartet, quin = quintet, sext = sextet, m = multiplet, brs = broad.

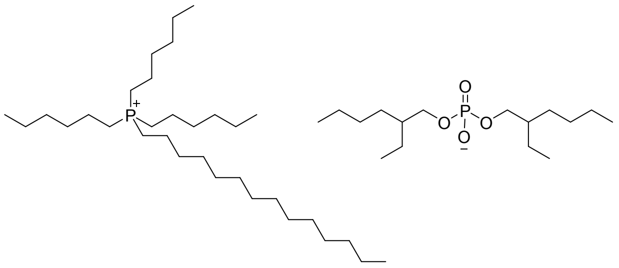

Figure S1. Structure of trihexyltetradecylphosphonium bis(2-ethylhexyl)phosphonate ($[P_{66614}]^+[BEHPA]^-$)

^1H-NMR (400 MHz, $CDCl_3$): δ_H = 3.70 (dd, 4H), 2.39 m (8H), 1.47 (m, 20 H), 1.27-1.21 (m,46), 0.85 (m, 24H). ^{13}C-NMR (400 MHz, $CDCl^3$): δ_C = 67.04, 40.55 , 32.00, 31.27, 30.94 (d, J_{P-H} = 15.0 Hz), 30.61 (d, J_{P-H} = 14.7 Hz), 30.20, 29.77, 29.73, 29.64, 29.44, 29.18, 23.42, 23.28, 22.78, 22.40, 22.06 (d, J_{P-H} = 4.6 Hz), 19.13 (d, J_{P-H} = 48.5 Hz), 14.24, 14.20, 14.04, 11.11. IR: ν(cm^{-1}) = 2956.0, 2923.8, 2855.5, 1462.0, 1378.5, 1244.5, 1044.6, 973.1, 810.3, 723.2.

Quadrupole ICP-MS method

Comparative measurements were done on an iCAP Qc quadrupole ICP-MS instrument (Thermo, Germany) using a polymeric concentric nebulizer, a glass cyclonic spray-chamber, and a quartz injector tube of 1.5 mm inner diameter. Other conditions were applied as summarized in table S1. Samples were taken up with an ESI SC2-DX autosampler in combination with an ESI FAST sample loop of 1 mL volume. At the beginning of the measurement session, the instrument was tuned for maximum sensitivity (^{115}In), as well as for low oxide ratios (CeO/Ce). The KED modus was operated with an energy barrier of -3V, and a mixture of hydrogen (7%) in helium was used as KED gas.

Table S1. Instrumental parameters used for ICP-MS measurements.

**Plasma power**	W	1550
**Cool gas (Ar)**	L min^{-1}	14
**Nebulizer gas (Ar)**	L min^{-1}	0.95
**Auxiliary gas (Ar)**	L min^{-1}	0.8
**KED-gas (H_2/He)**	mL min^{-1}	4.5
**Sample flow rate**	mL min^{-1}	0.5
**Cone material**	Nickel	
**Dwell time**	s	0.01
**Nuclides monitored**	^{45}Sc, ^{89}Y, ^{139}La, ^{140}Ce, ^{141}Pr, ^{143}Nd, ^{147}Sm, ^{151}Eu, ^{155}Gd, ^{159}Tb, ^{163}Dy, ^{165}Ho, ^{166}Er, ^{169}Tm, ^{172}Yb, ^{175}Lu; ^{115}In (internal standard)	
